# The Future High Education Distance Learning in Canada, the United States, and France: Insights From Before COVID-19 Secondary Data Analysis

**DOI:** 10.1177/0047239520940624

**Published:** 2020-12

**Authors:** Marguerite Wotto

**Affiliations:** 1 Interdisciplinary Research and Development, Center for Lifelong Learning (CIRDEF-UQAM), University of Quebec in Montreal

**Keywords:** distance learning, mobile learning, higher education, MOOCs, internationalization, lifelong learning

## Abstract

Evolving information and communication technology creates new spaces, learning materials, and demands in training institutions. Higher education distance learning (HEDL) responses to these transformations are miscellaneous and its development strategies vary from a country to another. Interpreting before COVID-19 secondary data, this article redefines the concept of distance learning and analyzes HEDL supply in Canada, the United States, and France. It enlightens its main current trends and challenges.

The COVID-19 crisis is triggering the online learning outbreak. We do not know what will remain when the crisis is over. If we must consider the data projected before the crisis, how can we see the evolution of distance learning in universities? Technological transformations will continue to grow around the world (Docebo, 2016; Organisation for Economic Co-operation and Development [OECD], 2017a), changing not only the landscape of trade and labor but creating news training and learning situations as well.In fact, they influence the accessibility and availability of distance education and training. For example, more than 4.4 million learners are enrolled in more than 2,497 programs and 18,342 courses in all disciplines at 27 open universities in the Commonwealth, spread over four continents (Africa, Asia, Europe, and America) with 300% growth in 2017 compared to 1987 (Commonwealth of Learning, 2017). These transformations intensify empowerment in learning, creating new relationships to knowledge, generating genuine needs and forms of learning, and requiring new ways of achieving this learning. More than access, education must focus on quality and learning relevance (Peña-López, 2015) to ensure a changing labor market competitiveness and the overall country economic performance (Zhang et al., 2017); develop adaptive postsecondary distance learning (DL) supply; and implement interventions that strengthen digital and technical skills, STEM, and employability skills (complex problem solving, critical thinking, creativity, management, etc.; World Economic Forum, 2018). The objective of this study is to analyze higher education distance learning (HEDL) supply in the three target countries and recent developments of HEDL.

## The Distance Learning top rated : Evidence from the Market 

 Social distancing has forced the change of any mode of learning in DL that increases unexpectedly during the COVID 19 pandemic. Either way, the DL was experiencing a deep change suggesting its growing importance in the market.

According to the litterature, DL faces several challenges including the emergence and rapid growth of learning needs, appropriate training delivery, and the supply adaptation to technological advances. Opposing Ambient Insight Research’s 2021 projections of a declining training market, technological change is creating new needs, and it uses and applications in education and training. The DL market generated revenues of US$42.7 billion in 2013 and US$46.7 billion in 2016 (Docebo, 2016).

**Figure 1.**
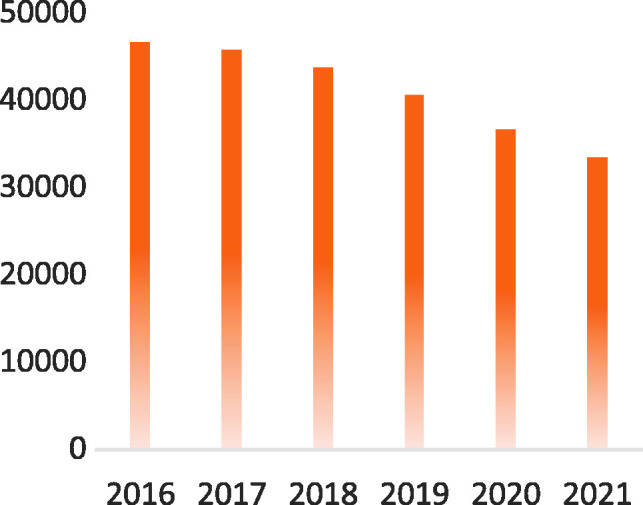
2016–2021 Worldwide Revenue Forecasts for Self-Paced e-Learning Products and Services (US$ Millions). *Source.* Docebo (2018).

**Figure 2.**
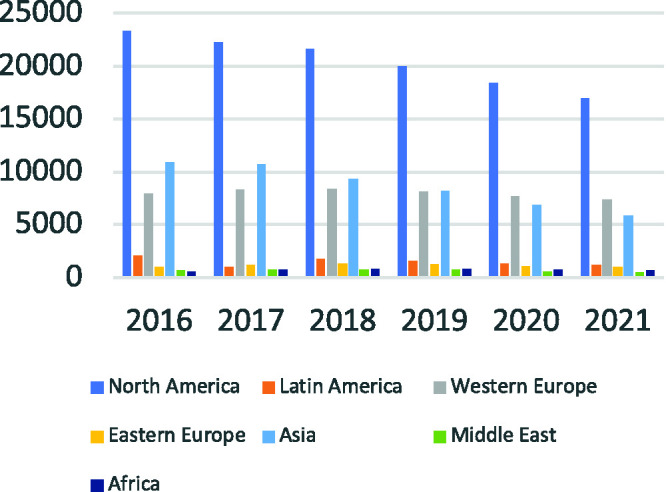
2016–2021 Worldwide Revenue Forecasts by Region (in US$ Millions). *Source.* Docebo (2018).

Between 2013 and 2018, it grew globally in all other World regions: Africa, 16.4%; Latin America, 9.7%; Asia, 8.9%; Eastern Europe, 8.4%; Central Europe, 6.3% (Ambient Insight Research, 2014 in OECD, 2015). With a projected annual growth of 10.26% between 2018 and 2023, it represents US$286.62 billion. Considering advanced improvements in artificial platforms and strong demand for flexible learning technology solutions, its revenue for 2021 is estimated at US$1,189 million in Latin America, US$16,967 million in North America, US$5,874.8 million in Asia, US$8.4 million in Europe, and US$636.3 million in Africa (Docebo, 2018; Figures 1 and 2).

Technological applications offer evolving and emerging learning opportunities (virtual classes, mobile learning, rapid e-Learning, etc.). Three quarters (74%) of the world’s population currently has access to email and each person will be connected to at least three devices by 2022 (OECD, 2019a). Internet use among 16- to 24-year-old is around 100% in most OECD countries (90% in Israel and Italy, 85% in Mexico and Turkey), higher among those with postsecondary education (OECD, 2017a). With the growing popularity of online learning among Generation Z and millenniums (Docebo, 2018), academic leaders consider DL as a growing force (Allen and Seaman, 2014 in OECD, 2015). In 2013, about 90% of American universities leaders indicated that a majority of students were likely to enroll in, at least, one online course within the next 5 years; 70.8% compared to 8.6% in 2014 of these leaders (Allen & Seaman, 2015) consider DL as critical to their institutions long-term strategy. This vision of DL should be supported by the national education system for the DL global competitiveness, the balancing of DL organizational objectives with learner practical learning needs.

Several studies highlight the correlation between the environment and learning outcomes. Consequently, DL investments and solutions should maximize learning spaces to increase any benefits related to this type of training (Barrett et al., 2019). For the majority of Canadian postsecondary institutions (69%), DL is important for their future, regardless of sector; more than 60% of the institutions with 30,000 or more students believe it promotes education innovation; one fifth see it as a way to implement provincial government policies (Bates et al., 2017a).With increasing automation and artificial intelligence on the verge of the fifth mobile generation, technological applications offer multiple potentials for innovation and training environment development. Rapidly evolving higher education DL models offers comparative advantages (Huynh et al., 2003; Wagner et al., 2008). To understand how DL is adapted to actual needs, national HEDL system could be understood by its supply transformations in the last few years.

## The Study Objectives

This study considers that any efficient response should take into account the technology use trends, the technological environment, and the learners’ habits and customs. It aims to answer the following fundamental question in consideration for the analysis: How do the national systems of the three countries respond to this growing demand? For doing so, it examines two subquestions: What are the changing trends in distance learning? How do national strategies align with these trends?

## The Methodology

This research is descriptive and uses secondary data. To analyze DL supply in the three countries, the adopted methodology approach is a systematic review of the literature based on flowing selection criteria: the research strategy, the study design, documents and report date of publication, and the quality of the documents. Data from the Google Scholar database and the websites of key stakeholders of the distance training (educational institutions, associations, and organizations) have been analyzed.

## Conceptual Framework

DL is an umbrella concept with a rich vocabulary, technical, varied, evolutionary, and encompassing several situations of equally different training. For Basak et al. (2018), digital learning brings together teaching technical solutions and learning in a format that fits today’s digital world of work and learning. Thus, DL should be constantly redefined to include a variety of emerging devices and practices. It includes online learning and all forms of education delivery to off-campus students (Bates et al., 2017a, 2017c), based on any training approach that replaces face-to-face in a traditional classroom in terms of specific time and place (Volery & Lord, 2000) for autonomous learning and requiring rare physical encounters between learners and their teachers (Ferreira, 2006). It is an educational process of teaching–learning (A. Martel, 1999), which implies, to a certain degree, a dissociation of teaching and learning in space and/or time (Conseil supérieur de l’éducation, 2015a, 2015b), volitional control of individual learning, noncontiguous communication between learner and trainer (Sherry, 1995), and technology (online or off-line).

Learning, which also includes teaching and its corollary training, could be defined as a process for the communication of instructive information between two parties (teacher trainers and learners) and that allows a learner to build required knowledge. It includes courses, programs, and other educational experiences delivered through traditional means (print, paper, and radio; Sherry, 1995) and/or online and within the entire spectrum of providing remotely instructive information (Sener, 2010). Even through media devices, the act of communication includes sometimes two-way exchanges between learners and trainers or with peers. In the context of widespread 21st-century internet use, is there a distinction between “remotely” or “online”? Many online tools (email, attachments, online review, viewing, printing, database, syllabus image, etc.) are used synchronously or asynchronously, both in the classroom and remotely by learners and teachers. This leads to blurred boundaries between these two modes of training generating a hybridization or creating a continuum between DL and traditional training (Charlier et al., 2006).

Online training provides access to educational experiences (Carliner, 2004; Conrad, 2002; Moore et al., 2011).However, with the internet, technological distance denies time and space referring to temporal/timeless, spatial/a-spatial, training/learning, and synchronous/asynchronous (Massé et al., 2014 in Grégoire, 2017). Thus, the various names such as distance education, multimedia training, open and distance learning, tailor-made training, e-learning (or e-training), and online training represent the same reality as DL (Deschênes & Et Maltais, 2006).

DL is formal when it refers to a set of activities organized in the education system (public or private educational institutions, colleges, universities, and other educational institutions), which are the normal pathway to full-time or part-time student enrolment (OECD, 2017b). It is framed by the demands and constraints of pedagogy and, even more, the transformation of training paths (A. Martel, 1999), the institutional accreditation of training, and the social recognition that accompanies it. The articulation of teaching/learning at the heart of the transmission/acquisition of information translates into another, that of the supply/demand for training. Demand is defined by targets, their representativeness, and their characteristics (European Commission/EACEA/Eurydice, 2018) including ease of use of mediated communication, perceived utility, and cost-benefit of training (Ferreira, 2006).

Chitkushev et al. (2014) consider DL as an educational tool which influences higher education offer in different ways (popularity, way of delivery, and it continues to get widespread and to gaining popularity day by day in the digital world. The offering is characterized by educational performance, success rate, external quality assurance, and recognition of informal and nonformal learning and its social dimension (European Commission/EACEA/Eurydice, 2018.). It depends on its funding and its capacity for systematic self-organization that needs to be captured from a holistic perspective of the change process, the emerging properties of creating and controlling technological change (Wotto et al., 2017), the institutional-level digital training strategy, leadership (Ngamau, 2013), the technical infrastructure (Masoumi, 2010), and management support (Mavengere & Ruohonen, 2010; Ngamau, 2013). It is influenced by institutional, managerial, and ethical factors (Basak et al., 2016), socioeconomic factors (Van der Wende, 2003), cultural factors, the education system and its institutional organization, and the changing role of government policies (Middlehurst, 2001).

## National Education System Responses to DL Needs

In the three countries, HEDL is characterized by a rapid increasing of registrations, the existence and spread of national network of suppliers giving birth complex platforms of DL (exceptionally in Canada and the United States), and internationalization, especially in France and United States. In these systems coexist accredited DL and Massive Open Online Courses (MOOCs).

### Higher Education DL in Canada

According to data from Bates et al. (2017a, 2017c), between 2011 and 2015, the Canadian higher education DL experienced an overall increase of 58% (about 11% per year) in enrolment in online courses; in 2015, these registrations accounted for about 16% of total registrations at Canadian universities. Between 2015–2016 and 2016–2017, more than 65% of institutions experienced a growth of more than 10% in online course registrations over the previous year; in 2016–2017, 236,917 higher education students or 67% of students were taking courses online; 75% of institutions indicated that they expected enrolment to increase (Bates et al., 2019). In 2017, 8% of all registrations for credited courses were fully online, representing just over 1.3 million online registrations. Therefore, there is still a lot of room for growth, although, for most campus institutions, it is unlikely that it will far exceed 20% of all course registrations (Bates et al., 2019).

According to Bates et al. (2017a, 2017b), the number of establishments transitioning to online training has increased by about 2% per year. DL is present in almost every Canadian university and most institutions have a good experience in Bates et al. (2019). DL accredited is provided by 98.1% of universities; over 6 years, a growth of 6% is mainly driven by medium-sized institutions (between 10,000 and 20,000 students) which make up half of the institutions that offer 87% of online courses. The average number of courses is almost the same in these institutions as in those of over 30,000 students. In addition, 824 additional DL courses are offered per year in Canada. This represents an average of 15 additional courses per institution; 87% offered hybrid courses. Moreover, 97% of Anglophone institutions offered online courses in 2016, compared to 61% of French-language institutions, but institutions offer at least three times as many credited courses as bilingual institutions (Bates et al., 2017a, 2017b, 2017c). At the undergraduate level, Anglophones offer more courses than Francophones (an average of 146 vs. 114). At the higher levels, the opposite trend is observed (40 vs. 54). Quebec is the second-largest province in Canada DL, considering the average number of distance learning programs per institution and fifth in terms of the average number of international students enrolled.

The internet remains the widely used technology (98% of institutions). Canada university DL offering is centralized in clusters of Canadian public institutions and common registration platforms—such as OntarioLEARN, eCampus Ontario, Contact North (Ontario), eCampus Alberta, BCampus (British Columbia), and Virtual University of Canada (UVC)—a consortium of Canadian universities in which Athabasca University is demonstrating its leadership.According to Bates et al. (2017a, 2017b) report, the majority of DL in the public sector is offered in disciplines such as administration, education, health sciences, information and technology, and community services.

Canada, institutions’ views on the future of MOOCS are highly diversified, with almost one third of institutions either having no interest in them (32.60%) and or believing existing MOOCs should be supported. Despite this limited enthusiasm for MOOCs, there is only an average of eight nonaccredited courses per institution. These courses are frequent in the francophone institutions landscape. Moreover, 50% of French-Canadian institutions already offer (or plan to offer) one or more MOOCs. In 2014, all French-Canadian institutions already offering MOOCs reported having completed one or two, with the exception of EDUlib, which offered 12. However, Anglophones are more active than Francophones in offering noncredited courses (8 vs. 28).

### The American Higher Education DL

With overall DL revenues of US$46.6 billion in 2016, North America will likely experience significant growth between 2016 and 2023 (Ambient Insight Research, 2016). Based on data from the U.S. Integrated Postsecondary Education Data System (IPEDS), enrolments are globally increasing mainly in management and business, social sciences, education and training, medicine and health, and engineering and technology (Allen & Seaman, 2017). However, there is very low enrolment in journalism and media, agriculture, and forestry. More than 5.8 million students took at least one course online in 2014; this represents 28.4% of all students enrolled compared to 27.1% in 2013 and 25.9% in 2012. Of these, more than 2.8 million students were enrolled exclusively in online courses. According to the same data, more than 147,169 new registrations took place in public higher education institutions in 2014. In general, 67% of registrations took place in public institutions and 33% in private for-profit and nonprofit institutions. Considering enrolments by cycle, 61% of graduate students are enrolled in private institutions (36% in nonprofit ones, 25% in lucrative ones) and 39% in public institutions. Of the total number of registrations in distance training, 27% were enrolled in the first cycle in private institutions, of which 12% is in nonprofit institutions and 15% in private for-profit institutions. Public institutions count for 73% of total registrations (Allen & Seaman, 2017). The majority of enrolments are in institutions with 1,000 or more students. Smaller institutions receive less than half (48.8%).

For Seaman et al. (2018), the total number of students enrolled in on-campus courses decreased by more than one million (1,173,805). This represents a decrease of 6.4% between 2012 and 2016. The largest decrease came from the private sector, which experienced a 44.1% declines over the period, while nonprofit institutions experienced a 4.5% decrease and public institutions 4.2%. The number of students who do not take distance courses declined by 11.2% (1,737,955) between 2012 and 2016. The for-profit private sector lost 50.5% compared to the nonprofit sector (9.5%) and public institutions, a decline of (7.7%).

Since 2015, the MOOCs have been dominated by traditional teaching in the United States. U.S. platforms, through varied geographical university partners, deal with the most prestigious institutions. More than 70% MOOCs are hosted by Coursera and 60% of those available on EdX are produced by universities included in the Shanghai Top 150 ranking; 70% of those registered on these platforms reside outside the U.S. territory (Delpech and Diagne, 2016.). The survey designed, administered, and analyzed by the Babson Survey Research Group, with additional data from the National Centre for Education (Education) and the Statistics IPEDS, indicated that several researchers consider that 11.3% of responding institutions offer MOOCs in 2015 compared to 8.0% in 2014 (Seaman et al. (2018)).

### France Higher Education DL

In recent years, French HEDL has especially favored centralization, promotion of MOOCs, and internationalization. It is mainly offered in engineering and business schools; law/economics/management disciplines, it is a niche offering of excellence courses in the second cycle where it represents 40% of the supply. The French Fédération interuniversitaire de l'enseignement à distance (FIED) brings together 35 of the 85 universities and has 30,000 students each year for bachelor’s and master’s degrees in almost all disciplinary fields. According to data from France Stratégie, the French DL has many facets, with its franchises, satellite campuses, and associated institutions.

France exports knowledge to more than 600 international programs on international campuses, including 330 outsourced graduate programs and 138 DL programs that reach nearly 37,000 students worldwide. Internationalization is also observed through the MOOCs. The country has developed its platform France Université numérique (FUN) under the aegis of the Ministry of Higher Education and Research. According to Delpech and Diagne (2016), FUN hosts more than 140 MOOCs followed by more than 500,000 registrants in France and abroad. The authors stress that the French strategy is to develop a potential market of 400 million students by 2030 to catch up with the Anglo-Saxon supply. This would be achieved through supply diversification defined by:

1. Income from new markets, in particular, continuing vocational training for employees of enterprises;
2. Offer to meet the needs of different audiences, giving priority to the certification and customized MOOCs;
3. A geographical customization that led, at the end of 2015, to more than 500,000 subscribers on the FUN platform which 70% in France on national MOOCs. This result represents a reversal trend since MOOCs generally serve 70% of international students. Only the universities of Paris Sorbonne and François Rabelais de Tours are present on the European site.

## A Business Model Offering a Greater Openness to Monetization and Certification

### Comparative Analysis of Higher Education DL System

Data highlight that high education DL is expanding in the three countries by undergoing several transformations and integrating technological evolution. Comparing enrolment rates in Canada in the fall of 2016 (Bates et al., 2017a, 2017c) with those in the United States at the same time (Seaman et al., 2018), it is observed that Canada HEDL enrolment exceeds that in the United States (Seaman et al., 2018; Bates et al., 2017a, 2017b, 2017c). In the United States, the majority of students (55%) taking distance courses in 2012 resided in the same territory of residence of their platform (Seaman et al., 2018). However, considering overall growth in enrolments in Canada and the United States, it is rather a decrease. In these two countries, it is difficult to say, which audiences are involved globally in the national DL. Table 1 presents summarizes the main characteristics of national HEDL in terms of supply.

**Table 1.** Characteristics of the HEDL in the Three Countries.

Countries	Higher education distance learning
Canada	Public Programs and courses in continuity with the face-to-face programs Adapted to its context Development more oriented towards the internal and external market Numerous actors The logic of centralized and partnership dominant organization Use of various technical means, but internet priority
United States	Public, private for-profit and private not-for-profit Programs and courses developed for HEDL Strong HEDL specialization The logic of dominant delegated organization More domestic- and international-oriented development Strong adaptation Strong MOOCs growth—FUN platform
France	Public, private for-profit and private not-for-profit Programs and courses developed for HEDL Strong HEDL specialization The logic of predominant organization mutant towards centralization Adapted to its context More domestic- and international-oriented development Strong adaptation Strong growth in private for-profit MOOCs (leadership)

*Note.* HEDL = Higher education distance learning; FUN = France Université numérique; MOOCs = Massive Open Online Courses.

Several universities in the three countries consider DL adoption as a training diversification strategy to ensure profitability and consolidate its position in education. There is also a strategy of specialization without expanding the market: This is the case of Canadian universities, which primarily satisfy the national market. It is observed that in Canada, surveys considered in this literature review demonstrate a priority in meeting national needs with existing on campuses courses and programs completed with DL. It is a matter of either centralization of services or very autonomous university strategies. Table 1 shows the main characteristics of the HEDL in the three countries.

A growing supply that looms in continuity training and diversifies from the community-specific needs requires a service consolidation. As Bates et al. (2019) points out, MOOCs are an interesting and useful development, but they have moved into a niche for continuing and in-company training rather than disrupting the current system.

The development of unaccredited higher education DL in Canada appears to be in contrast to that observed in France and the United States. In France, the government strategy encourages the development of MOOCs. The “MOOCization” focused on diversifying international and professional markets in the workplace. In the United States, DL shows a decline leading to the development of private for-profit MOOCs platforms based on a delegated logic.

The fundamental differences between Canada, France, and the United States higher education DL could be expressed in terms of hybridization, internationalization, level of development of MOOCs, strong centralization (FUN platform) private sector participation, and functional delegation.

As Bates et al. (2019) points out, MOOCs are an interesting and useful development, but they have moved into a niche for continuing and in-company training rather than disrupting the current system. MOOC adoption is considerably higher in the U.S. institutions in 2013 and 2014 (14%), but lower in Europe, at 72% in 2014. In 2017, Bates et al. (2019) found that there is not much interest in open-to-all online training (MOOC) in Canada. Fewer than 20% of the responding institutions had offered them in the past 12 months, and those offering only a few courses. One third (32.60%) of Canadian institutions have no interests in MOOCs. In contrast, in 2014 in the United States, 51% of respondents disagreed with this statement, perhaps because it was still early to decide; 11.3% of institutions offered MOOCs in 2015 (8.0% in 2014, in 2012: 2.6%, 2013: 5%, 2014: 8.0%). Fewer than 20% of the responding institutions had offered them in the past 12 months, and those offering only a few courses. In his survey, Grégoire (2016) stated that 50% of French-Canadian institutions already offer (or plan to offer) one or more MOOCs, a rate considerably higher than in the U.S. institutions in 2013 and 2014 (14%), but lower than Europeans (72% in 2014).

## Discussion

Several reasons and strategies contribute to DL development in the three countries. However, this consistent transformation of HEDL following the digital transformation put in shadow four transformations: MOOC explosion, mega portals birth, DL internationalization, and the mobile and lifelong learning. These transformations expose national HEDL to emerging challenges, which should call for enhancing higher education strategies.

### The MOOC Explosion

As Delpech and Diagne (2016) points out, MOOCs have grown exponentially from 10 in 2011 to thousands today. The MOOC market represents today a luxuriant market that offers various digital platforms in the world. Some key players with different business profiles and strategies such as Pluralsight (United States), Edureka (India), Alison (Ireland), Udacity (United States), Udemy (United States), Miríadax (Spain), Jigsaw Academy (India), Simplilearn (United States), Iversity (Germany), Intellipaat (India), Edmodo (United States), FutureLearn (UK), LinkedIn (United States), NovoEd (United States), Open2Study (Australia), WizIQ (India), Skillshare (United States), XuetangX (China), Federica (Italy), Linkstreet Learning (India), Khan Academy (United States), and Kadenze (Spain) are offering recent development and competitive advantage. Except for German Iversity, these platforms have a very low offer from other European countries institutions. FUN’s offer is almost 98% from French institutions compared to national content with MiriadaX (75%) and FutureLearn (66%). The United States hosts the highest number of MOOC platforms. In Europe, Spain is the country with the largest number of 505 MOOCs. It is followed by the United Kingdom. Italy has the lowest number of 96 MOOCs. Several U.S. platforms are private and for-profit. However, the American edX platform like the French FUN is not-for-profit. Moreover, 3% of universities in France against 80% in the United States put their courses online. According to data from Open Education Europa (in Delpech & Diagne, 2016), the edX platform has 5 million registrants, FutureLearn 2.5 million of which almost two thirds are out of the country.

MOOCs are a showcase to promote higher education institutions, particularly abroad, and to reach new audiences through more flexible and personalized training offerings (Delpech & Diagne, 2016.). Although they can play a formative role in higher education, they miss encouraging long-term personalized learning, training strategies, and accreditation or certification forms. Due to the massive participation, the high heterogeneity of participants, the lack of target groups, and the varying commitment of learning, Henri (2017) considers that analytic and prescriptive rigor of pedagogical engineering seems difficult to apply to the design of MOOCs. Except in France, countries miss a national business and monetization model, adaptive learning, and learning recognition framework. As stated by Kiers and van der Werff (2019), HEDL needs an operational model, which requires obtaining and implementing insight into factors in MOOC-based programs.

### Megaportal and HEDL Internationalization

While international student enrolment continues to rise at U.S. universities (Hellmann & Miranda, 2015), HEDL internationalization takes place through mega portals such as the Study Portals. This portal offers more than 170,000 courses from 3,050 educational institutions in 110 countries, 12,698 programs, including 2,464 bachelor’s degrees, 6,475 master’s degrees, 470 doctorates, and 2,998 short programs. Its partners include British Council, European Commission European Commission, Nuffic, German Academic Exchange Service (DAAD), Austrian Academic Exchange Service, Universidades in Spain, Academic Cooperation Association, and pan-European network of several nonprofit organizations, responsible for the internationalization of education and training such as UNESCO Institute for Lifelong Learning, a nonprofit organization, EADTU, Cambridge Assessment English, a division of Cambridge University, International Council for Open and Distance Education (ICDE), Swedish Institute, Open Education Europa, and so forth. In 2017, Studyportals reportedly helped more than 28 million students around the world explore curricula and make an informed choice. According to its website data, the megaportal registered 195,400 international student registrations in 2016. The number of registrations grew by 28.40% in 2014, while in 2012 this growth was 25.90%. The profile of the typical student is 51%women and 49% men. It offers programs in agriculture and forestry, applied sciences, art, design and architecture, management and business, computer and information technology, education and training, engineering and technology, Environmental Studies and Earth Sciences, Recreational Hospitality and Sport, Humanities, Law, Journalism and Media, Medicine and Health, Natural and Mathematical Sciences, and Social Sciences.

Paralleled to megaportals, higher education internationalization has become, in many countries, a major expansion issue and enrolment growth target. More than program outsourcing, online learning contributes to increasing enrolment providing access and flexibility. Learners from all over the world increasingly invest time and money for personal progress. In the same time, HEDL in national education remains an extension of in-class education. Furthermore, it is limited to a few programs. In Canada, Bates et al. (2019) note that at least half of universities offer online programs in administration, arts, humanities, and education. However, none of the universities surveyed offered programs in dentistry, engineering, forestry, medicine, or pharmaceutical sciences. Bates et al. (2019) point at many reasons—the lack of appropriate staff to develop and deliver these courses or the additional faculty effort required to develop or deliver online courses—which were similar to the national response. In the United States, enrolments are mainly in management and business, social sciences, education and training, medicine and health, and engineering and technology.

### Mobile and Lifelong Learning Matter

If Higher Education needs to promote lifelong learning, strategic transformative approaches should not only broaden access but also increase learning quality and learner satisfaction (Yang et al., 2015). Despite the increasing enrolment in the United States, there has been a growing decline in student satisfaction: from 92% in 2012–2013 to 86% in 2016. The emerging issues in DL are social learning, mobile learning, micro learning, and learner skilling in organizations. Recent Docebo (2018) surveys noticed that 53% of learners mentioned location as a barrier to online learning. So they turned to mobile learning. In addition, 64% of learners declared that learning on a mobile device is essential or very useful; they access their training content from a mobile device essential. Smartphone learners complete course material 45% faster than those using a computer; 89% of smartphone users download apps, 50% of which is used for learning; 43% learners see improved productivity levels compared to nonmobile users; 71% of Millennials say they connect more with mobile learning than L&D activities delivered via desktop or formal methods; the number of mobile-only users (27%) has grown, now surpassing desktop-only users (14%); 46% of learners use mobile learning before they go to sleep at night. In French-speaking Canada, a survey showed 4 out of 5 participants say that the massive mobile devices use in their community has had an impact on their way of teaching.

As Merhan (2017) points out, another dimension to consider is now a matter of adopting a HEDL vision that is more concerned with professionalization, upskilling, reskilling for the market increasing demand for competences, and curricula adaptation. For this purpose, all the three countries market still has high potential from a vocational and business training perspective. That hypothesis should be tested. Furthermore, the learner’s commitment to learning must be linked to a personal project. Carré (2014) promoting a high-quality, equitable and global learning experience will help graduates to prepare and contribute to a globally interconnected society. Points out that the starting point lies in the analysis of the individual conditions of learning and skills development. For the author, training performance exists in underestimating the learner's logic. If online learning provides better access and flexibility for students, comparability between education systems and the transferability of qualifications obtained by HEDL are prerequisites for improving student mobility (Henri, 2017) and for developing their global competency.

Furthermore, the reality of today’s learning and training is no longer limited to institutionalized education. Adding new subjects or learning areas to the taught curricula traditionally designed around specific disciplines and/or learning areas can lead to curriculum overload, while embedding them within existing subjects can prove challenging, given the conceptual complexity of some of these competencies (OECD, 2019b). As the rapid labor market transformations challenge societies and individual, lifelong learning becomes the foundations of providing continuous upskilling and reskilling learning. It promotes adaptation to leaning and full work participation for core skills, knowledge, attitudes, and values that are prerequisites for further learning across the entire curriculum (OECD, 2019b). LLL contributes also to the continuous professional development of the active population, thus improving autonomy and internal flexibility (Wotto et al., 2017). LLL vision could help to motivate MOOC learners, to better identify and understand how the uses of MOOC may or may not participate in producing inequality (Vayre & Lenoir, 2019), to show how the uses that e-learners make of this course are shaped by the weight of social structures. Finally, through HEDL transformation, it becomes important to clarify the “different potential for technological deskilling/upskilling, namely the ability of ICTs to contribute to the moral deskilling of human users” (Vallor, 2015,  pp. 107-124).

## Conclusions and Limits

The national higher education DL is adapting supply to technology transformations dominated by many forces, namely, service centralization and internationalization. In the United States, both public and private (for-profit and not-for-profit) institutions support these forces. In France, the national strategy is especially oriented to abroad. In Canada, institutions develop their own HEDL strategy. The study shows that the DL in Canada is growing to assume the coverage of the national territory, but for organizational effectiveness (institutional performance). However, like France, Canadian institutions must catch up on the international stage. However, HEDL development does not follow the tendencies of the learners and DL market trends. MOOC development faces a lack of business model. The DL sometimes follows the traditional education because of lack of development new training strategy. The interest for HEDL internationalization is growing for training institutions leading to megaportals development. Although the growth of digital platforms helps to concentrate many services, quality, credibility for organizations and skills, and employability matter for individuals should overcome any commercial reason. If m-learning can be capitalized to enhance quality and access and to help learning and future employability, training geared to the acquisition of renewed skills expressed in terms of professionalization also aims at self-efficacy in learning for the mobilization of skills in context. In a knowledge-based economy with changes in skills and occupational profiles, we are a long way from customizing training that requires learner-centered learning.

The comparison in this study should be understood in the context of this report, a context, which considered that the training system falls within a sociopolitical context with its norms, rules, and values to which we have not alluded in this report. Furthermore, this study presents the cumulative limitations of the studies and reports selected. One of its main limits is the secondary database analysis. Further research should provide evidence to understand the trends in learning and training and their impacts.
